# Online-Untersuchungskurs statt Präsenzveranstaltung: Anpassung der studentischen Lehre im Medizinstudium während der COVID-19-Pandemie

**DOI:** 10.1007/s00347-021-01372-x

**Published:** 2021-04-06

**Authors:** Cornelius Wiedenmann, Katrin Wacker, Daniel Böhringer, Philip Maier, Thomas Reinhard

**Affiliations:** Klinik für Augenheilkunde, Universitätsklinikum Freiburg, Killianstr. 5, 79106 Freiburg, Deutschland

**Keywords:** Medizinische Lehre, E‑Learning, Lehrevaluation, Kompetenzen, Video, Medical education, E‑learning, Teaching evaluation, Skills, Video

## Abstract

**Hintergrund:**

Da Präsenzveranstaltungen im Sommersemester 2020 durch die Coronavirus-Disease-2019(COVID-19)-Pandemie nicht möglich waren, entwickelten wir einen Online-Untersuchungskurs.

**Ziel der Arbeit:**

Die Studie untersuchte die Zufriedenheit der Student*innen mit dem Online-Untersuchungskurs und die Auswirkungen auf die praktische „Objective structured clinical examination“(OSCE)-Prüfung.

**Material und Methoden:**

Der Online-Untersuchungskurs wurde als videobasiertes Tutorial nach dem SMART(Specific, Measurable, Activating, Reasonable, Time-bound)-Prinzip entwickelt und allen Student*innen des Blockpraktikums Augenheilkunde zur Verfügung gestellt. Die Student*innen benoteten am Ende des Semesters den Online-Untersuchungskurs mittels Online-Fragebogen auf einer Ordinalskala von 1 bis 6 und wurden um Freitextkommentare gebeten. Die relative Punktzahl der OSCE-Prüfung am Ende des Semesters wurde mit den Prüfungsergebnissen des vorangegangenen Semesters verglichen. Alle Auswertungen erfolgten anonymisiert.

**Ergebnisse:**

Es beteiligten sich 67 der 164 Student*innen des Blockpraktikums an der Online-Evaluation (41 %). Die Student*innen fühlten sich auf die praktische Prüfung gut vorbereitet (Mittelwert 2,0; SD 1,0); 70 % der Student*innen (47 von 67) wünschten sich auch zukünftig die Möglichkeit eines digitalen Angebots in Ergänzung zur Lehre vor Ort. Die Prüfungsergebnisse im OSCE waren im Mittelwert mit einer Note von 1,1 sehr gut (SD 0,2; *n* = 164) und ähnlich zum vorangegangenen Semester (Mittelwert 1,1; SD 0,2; *n* = 166, zweiseitiger *t*-Test *p* = 0,86).

**Diskussion:**

Der Online-Untersuchungskurs erlaubt es den Student*innen, sich auf die OSCE-Prüfung vorzubereiten. Über die COVID-19-Pandemie hinaus kann die Bereitstellung eines Online-Untersuchungskurses eine gewinnbringende Ergänzung zur Präsenzlehre darstellen.

## Abstract (en)

**Background:**

The coronavirus disease 2019 (COVID-19) pandemic restricted face-to-face interactions of medical students and teachers.

**Objective:**

To develop and evaluate an online course for ophthalmology examination techniques for medical students and to determine the impact of the course on the practical objective structured clinical examination (OSCE).

**Material and methods:**

The online examination course was developed as a video-based tutorial according to the SMART (specific, measurable, activating, reasonable, time-bound) principle covering all topics of the National Competency-based Catalogue of Learning Objectives for Medical Education and made available to medical students in the ophthalmology course. At the end of the semester the students graded the online examination course on an ordinal scale from 1 (strongly agree) to 6 (strongly disagree) anonymously and were asked for comments. The grades of the OSCE (range 1–5) were compared with the OSCE results of the previous semester.

**Results:**

Of the 164 students 67 participated in the online evaluation (41%). The students reported to be well-prepared for the OSCE (mean grade 2.0; SD 1.0). In the future, 70% of the students would prefer combined online and on-site teaching (47 out of 67 students). Among all 164 students, the mean OSCE results were excellent (mean grade 1.1; SD 0.2) and comparable to the previous semester (mean grade among 166 students, 1.1; SD 0.2; two-sided *t*-test, *p* = 0.86).

**Conclusion:**

The online course on ophthalmology examination techniques allowed medical students to prepare for the OSCE. Beyond the COVID-19 pandemic, online teaching options can support on-site teaching.

Der praktische Untersuchungskurs ist wesentlicher Bestandteil des Blockpraktikums Augenheilkunde an der medizinischen Fakultät Freiburg. Er soll künftige Ärzt*innen aller Fachrichtungen dazu befähigen, Basisfertigkeiten wie das Abschätzen des Augeninnendrucks oder das Entfernen eines subtarsalen Fremdkörpers zu erlernen. Aufgrund der Coronavirus-Disease(COVID)-19-Pandemie waren Präsenzlehre im Sommersemester 2020 und damit auch der Untersuchungskurs in seiner bisherigen Form nicht durchführbar. Wir entwickelten daher einen Online-Untersuchungskurs, um die Basisfertigkeiten zu vermitteln.

## Hintergrund

Der Untersuchungskurs Augenheilkunde ist in Freiburg seit 2006 fester Bestandteil der Ausbildung angehender Ärzt*innen. Entsprechend den Zielen der Approbationsordnung für Ärzte werden hier „grundlegende Kenntnisse, Fähigkeiten und Fertigkeiten vermittelt, die für eine umfassende Gesundheitsversorgung der Bevölkerung erforderlich sind“. Entsprechend dem Nationalen Lernzielkatalog umfasst der Untersuchungskurs die Themen direkte Fundoskopie, Ektropionieren des Lids mit Fremdkörperentfernung, Palpieren des Augeninnendrucks im Seitenvergleich, Sehschärfenbestimmung, Pupillenreaktion, Applikation von Augentropfen und -salben und Spaltlampenuntersuchung der Bindehaut und Hornhaut. Lernziel des Untersuchungskurses ist es, die genannten Untersuchungsmethoden unter Anleitung selbst durchführen und demonstrieren zu können, was der Ebene 4A des Lernzielkatalogs der Deutschen Ophthalmologischen Gesellschaft (DOG) für Augenheilkunde entspricht [12].

Digitale Lehr- und Lernangebote in der medizinischen Ausbildung sind aktuell ausgesprochen heterogen. Die Formate reichen von reiner Aufzeichnung der Vorlesung zu speziell konzipierten Instrumenten des E‑Learning und werden auf unterschiedlichen Plattformen wie Webseiten (z. B. https://eyetube.net/, https://ophthalmo-update.com/) oder Apps (z. B. AAO Ophthalmic Education App, Eye Handbook, verfügbar in Google Play Store für Android und Apple App Store für iOS) angeboten. Allgemein gesprochen, können digitale Lehrangebote im Ergebnis ebenbürtig sein oder teils sogar bessere Lerneffekte erzielen [8]. Der Lernerfolg praktischer Fähigkeiten durch Videos ist schriftlichen Lernmaterialien teilweise überlegen [7]. Dies kann besonders in der Ophthalmologie, einem visuellen Fach, von Vorteil sein [16]. Die COVID-19-Pandemie und die damit einhergehenden Einschränkungen in der Klinik und der Präsenzlehre stellten die Dozent*innen der medizinischen Fakultäten vor die Herausforderung, die Vorgaben insbesondere hinsichtlich des „praktisch ausgebildeten Arztes“ zur erfüllen und „Fähigkeiten und Fertigkeiten“ zu vermitteln [18]. So beklagten Bartz-Schmid und Hörauf, dass studentische Untersuchungskurse den notwendigen Sicherheitsmaßnahmen zum Opfer fielen [4].

In dieser Arbeit entwickelten wir einen Online-Untersuchungskurs und evaluierten die Zufriedenheit der Student*innen mit dem neuen Lehrformat. Um objektiv die vermittelten Fähigkeiten und Fertigkeiten durch das Lehrformat zu untersuchen, verglichen wir die Noten der Student*innen des ersten Online-Untersuchungskurses mit den Noten des vorherigen Semesters. Ziel der Arbeit war es, die Vor- und Nachteile des Online-Untersuchungskurses zu evaluieren, um die Chancen dieses Formates zu erkennen und es weiter zu verbessern.

## Material und Methoden

### Entwicklung des Online-Untersuchungskurses

Der Online-Untersuchungskurs wurde im April 2020 an der Klinik für Augenheilkunde Freiburg entwickelt. Der Inhalt des Online-Untersuchungskurses sollte die identischen Themen des Präsenzuntersuchungskurses umfassen und entsprechend dem Akronym SMART gestaltet sein. Unter SMART versteht man auf Deutsch spezifisch, messbar, aktivierend, realistisch und terminiert. Spezifisch bedeutete, dass zu Beginn jedes Themas das Lernziel beschrieben wurde. Messbar sollte es sein, indem auf wichtige Teilschritte hingewiesen wurde, die relevant für die Punktvergabe in der „Objective structured clinical examination“(OSCE)-Prüfung waren. Aktivierend sollten die Videos sein, indem praktische Beispielsituationen beschrieben wurden, die für Mediziner*innen jedes Fachbereichs nützlich sein können. Terminiert wurde der Online-Untersuchungskurs durch den Termin der OSCE-Prüfung.

Es wurde eine Videothek mit 8 Videos erstellt. Darin wurden die Untersuchungstechniken des Präsenzuntersuchungskurses kurz und prägnant dargestellt. In einem ersten Einführungsvideo wurde der Aufbau des Online-Untersuchungskurses erläutert. Zwei Sprecher*innen führten durch die Beispielsituation und erklärten die Relevanz der jeweiligen Untersuchungen. Anschließend wurden sowohl die manuelle Technik als auch erforderliche Geräte detailliert aus unterschiedlichen Perspektiven demonstriert, und dann wurde die vollständige Untersuchung korrekt durchgeführt. Zusätzlich wurde auf mögliche Fehlerquellen hingewiesen. Um eine durchgängige, erklärende Tonspur über verschiedene Kameraeinstellungen und Sequenzen hinweg zu realisieren, wurden die beiden Sprecherstimmen im Nachhinein aufgenommen.

**Figure 1:**
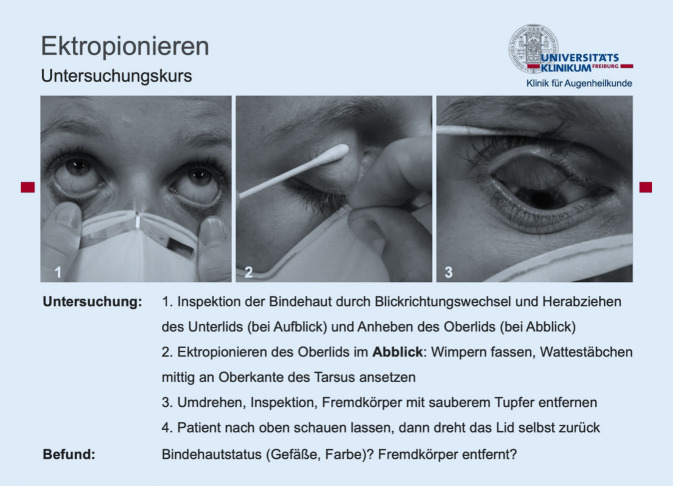

Weiterhin wurde ein Skript mit Abbildungen entwickelt, das die Untersuchungstechniken beschrieb und als „Spickzettel“ dienen sollte (Abb. 1). Im schriftlichen Dokument wurden die Untersuchungen schrittweise erklärt. Wichtige Teilschritte wurden als Fotografie ergänzend abgebildet. Zusätzlich wurde auf weiterführende Literatur verwiesen. Videos und Schriftdokument waren in Aufbau und Inhalt kongruent, sodass sie sich entsprechend der Flipped-Classroom-Methode ergänzten. Bei dieser Methode steht eine Selbstlernphase mit Skript vor dem eigentlichen Untersuchungskurs [5, 9, 17].

In einem abschließenden Selbsttest konnten die Student*innen überprüfen, ob sie wesentliche Inhalte verinnerlicht hatten. Schließlich wurden als Vorbereitung für die praktische Prüfung 2 einstündige Online-Sprechstunden mit dem Lehrbeauftragten als Online-Konferenz (Zoom, San Jose, CA, USA) angeboten, wo Fragen zu den verschiedenen Inhalten des Online-Untersuchungskurses diskutiert und beantwortet wurden.

### Durchführung der praktischen Prüfung (OSCE)

Die praktische Prüfung in Form eines OSCE erfolgt an der Klinik für Augenheilkunde seit 2006 und prüft die im nationalen kompetenzbasierten Lernzielkatalog und im Lernzielkatalog der DOG [12] genannten Kompetenzen an 4 von 6 möglichen Stationen. Die Stationen umfassen die Themen direkte Ophthalmoskopie, Palpation des Augendrucks, Ektropionieren mit Fremdkörperentfernen, Prüfung der Pupillenreaktion und Kataraktoperation. Die Inhalte des Untersuchungskurses und die zu erreichenden Kompetenzstufen sind in einem Qualitätsmanagementdokument festgelegt. Die fachgerechte Durchführung wird an jeder Station von je einer Prüfer*in nach vorgegebenen Kriterien mit 0 bis 20 Punkten bewertet, wobei jeweils ein Zeitlimit von 3 min besteht. Bedingt durch die Maßnahmen der Corona-Verordnung und Umsetzung an der Albert-Ludwigs-Universität wurde die Prüfung von 4 auf 3 Untersuchungstechniken gekürzt. Dabei wurde auf Ektropionieren und Fremdkörperentfernung verzichtet, da dies sich nur schlecht am Modell vorführen lässt. Die Reduktion von 4 auf 3 Untersuchungstechniken aus hygienischen Gründen führte zu einer Reduktion der Maximalpunktzahl von 81 auf 60 Punkte. Die erreichten Punktwerte und Noten des OSCE aus dem Sommersemester 2020 wurden mit den Ergebnissen des Wintersemesters 2019/2020 verglichen, indem prozentual der Anteil der erreichten Punktwerte den maximal erreichbaren Punktwerten gegenübergestellt wurde. Außerdem wurde der Unterschied der Prüfungsnoten der beiden Semester mittels zweiseitigen *t*-Tests überprüft.

### Evaluation des Online-Untersuchungskurses

Für die Evaluation des Online-Untersuchungskurses wurde der „Fragebogen für die digitale Lehre“ des Kompetenzzentrums Evaluation in der Medizin des Studiendekanats der Medizinischen Fakultät der Albert-Ludwigs-Universität Freiburg modifiziert und an den Online-Untersuchungskurs der Klinik für Augenheilkunde angepasst. Die Evaluation wurde in 4 Abschnitte aufgeteilt: 1. Formate digitaler Lehre (5 Fragen), 2. Gesamtbewertung der digitalen Umsetzung der Lehrveranstaltung (4 Fragen), 3. praktische Fertigkeiten (6 Fragen), 4. allgemeine Lehrkompetenz (5 Fragen). Die Student*innen des Blockpraktikums wurden gebeten, die Aussagen auf einer sechsteiligen Likert-Skala von „sehr gut“ bis „sehr schlecht“ und „trifft voll zu“ bis „trifft gar nicht zu“ zu bewerten und Freitextkommentare zu formulieren. Die Evaluation des Blockpraktikums war freiwillig. Die Student*innen wurden insgesamt 3‑mal vom Lehrbeauftragten um Teilnahme an der Evaluation per E‑Mail gebeten. Nach Beendigung der Umfrage wertete das Kompetenzzentrum Evaluation die Evaluationsergebnisse mittels EvaSys (Electric Paper Evaluationssysteme GmbH, Lüneburg) aus und stellte die Bewertungen als Mittelwerte mit Standardabweichungen und die Freitextkommentare anonymisiert zur Verfügung.

## Ergebnisse

Im Sommersemester 2020 nahmen 164 Student*innen am Blockpraktikum und der abschließenden OSCE-Prüfung der Klinik für Augenheilkunde des Universitätsklinikums Freiburg teil.

Die 8 Videos mit einer Gesamtdauer von 21:58 min (min. 1:06 min, max. 4:09 min) wurden jeweils durchschnittlich 268-mal (min. 224, max. 310) abgerufen. Laut eigener Angabe sah etwa ein Drittel der Student*innen (34 %, 23 von 67) die Untersuchungsvideos 2‑mal an und ein Drittel sah die Videos 3‑mal an (37 %, 25 von 67); 99 % der Student*innen berichteten, dass die Technik einwandfrei funktionierte (66 von 67). Das Skript zum Online-Untersuchungskurs wurde 356-mal heruntergeladen. Die Fragen zum Selbststudium wurden laut eigener Aussage von 91 % der Student*innen bearbeitet (60 von 66). Die Evaluation wurde von 67 Student*innen bearbeitet (41 %).

Das OSCE fand an 5 Tagen mit allen 164 Student*innen statt. Von 60 maximal erreichbaren Punkten wurden im Median 60 Punkte (100 % der maximalen Punktzahl) vergeben (Interquartilenabstand, 57 bis 60 Punkte). Im Wintersemester 2019/2020 wurden bei 166 Student*innen im Vergleich dazu im Median 78 Punkte (96 % der maximalen Punktzahl von 81 Punkten, Interquartilenabstand 76 bis 80 Punkte) vergeben. Die resultierenden Noten im Online-Untersuchungskurs waren im Mittelwert mit 1,1 sehr gut (SD 0,2; *n* = 164) und zum vorangegangenen Präsenzuntersuchungskurs (Mittelwert 1,1; SD 0,2; *n* = 166) nicht signifikant verschieden (*p* = 0,86).

**Figure 2:**
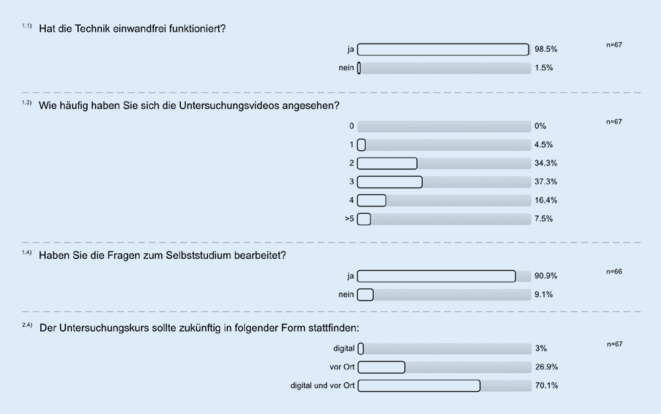

Eine deutliche Mehrheit der Student*innen wünscht sich zukünftig eine Kombination des Untersuchungskurses aus Digital- und Präsenzunterricht (70 %, 47 von 67). Nur 3 % der Student*innen würden einen rein digitalen Untersuchungskurs bevorzugen (2 von 67); 27 % der Student*innen hätten den Untersuchungskurs zukünftig am liebsten ausschließlich vor Ort (18 von 67) (Abb. 2).

Die Student*innen stimmten zu, dass der digitale Kurs wesentliche Untersuchungstechniken beigebracht habe (Mittelwert der Note 2,2; SD 0,9). Die zur Verfügung gestellten Dokumente wurden im Mittel mit der Note 1,8 (SD 0,8) und die Videos mit der Note 1,5 (SD 0,7) bewertet; 94 % der Student*innen stimmten der Aussage zu, dass sie sich anhand der zur Verfügung gestellten Materialien auf das OSCE vorbereiten konnten (63 von 67). Zurückhaltend waren die Student*innen bezüglich des Fachs Augenheilkunde als berufliche Perspektive mit einer durchschnittlichen Angabe von 3,6 (SD 1,4). Allerdings war die Streubreite der Antworten bei dieser Frage im Vergleich zu den anderen Fragen am größten; 25 % beantworteten die Frage mit voll zutreffend oder zutreffend (15 von 62).

**Figure 3:**
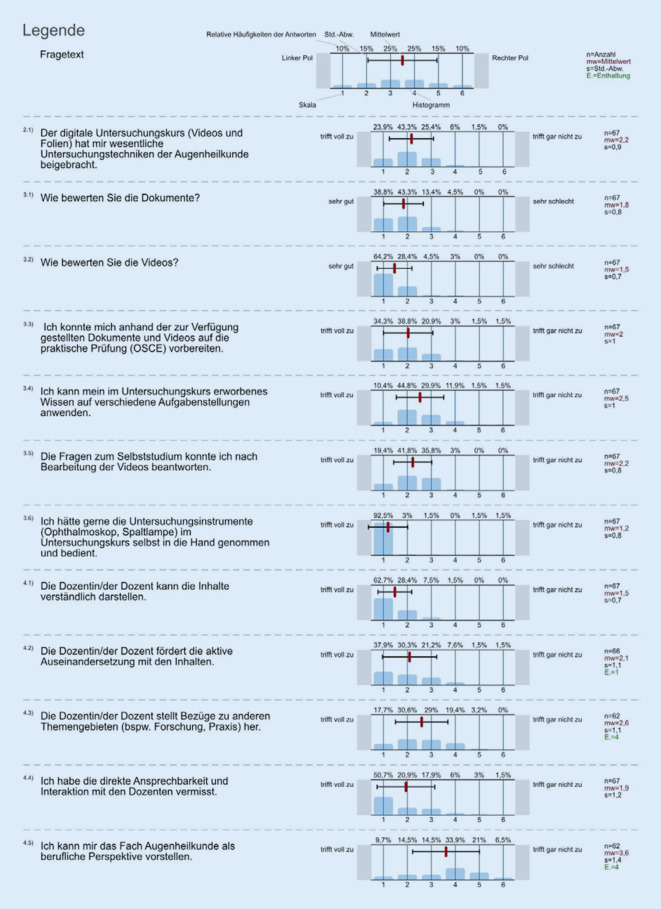

Die direkte Ansprechbarkeit und Interaktion mit den Dozent*innen wurde von den Student*innen vermisst (1,9; SD 1,2). Ebenfalls hätten die Student*innen gerne die manuellen Fertigkeiten an den Untersuchungsinstrumenten geübt (1,2; SD 0,8) (Abb. 3).

### Freitextkommentare

**Figure 4:**
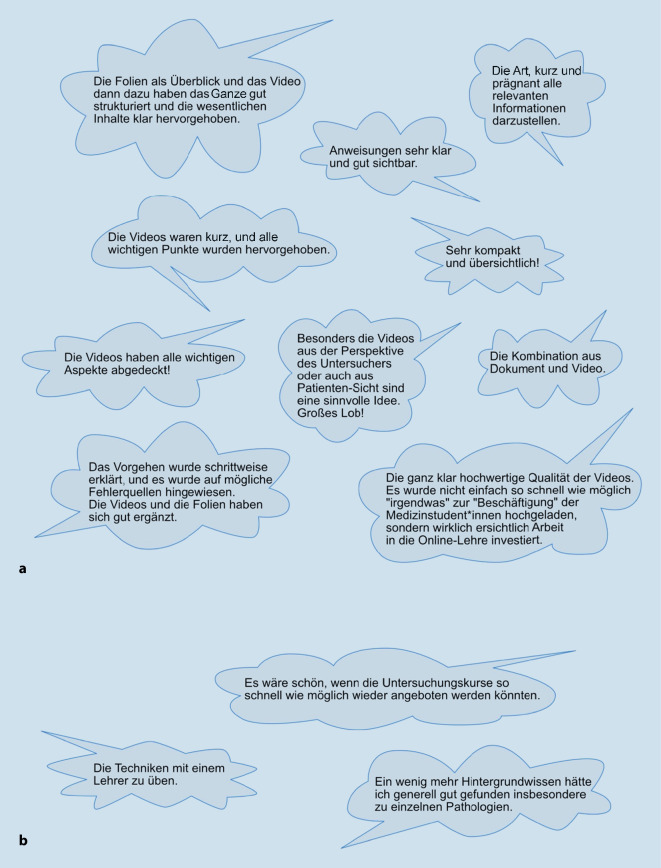

Auf die Frage „Was hat Ihnen bei den zur Verfügung gestellten Dokumenten und Videos gut gefallen?“ wurde die Prägnanz der Materialien wiederholt hervorgehoben. Auch die Qualität der Videos wurde hochwertig empfunden. Auf die Frage „Wo sehen Sie Verbesserungsbedarf bei den zur Verfügung gestellten Dokumenten und Videos“ wurden mehr klinische Beispiele gewünscht. Außerdem gaben viele Student*innen an, sie hätten vermutlich von praktischen Übungen mit den Untersuchungsinstrumenten profitiert. Eine repräsentative Auswahl der Freitextkommentare ist in Abb. 4 dargestellt.

## Diskussion

In der Evaluation des Online-Untersuchungskurses zeigte sich, dass eine Kombination aus bebildertem Skript und Videos den Student*innen wesentliche Untersuchungstechniken der Augenheilkunde beibringen kann. Auch wenn die persönliche Interaktion in der Präsenzlehre vermisst wurde, sahen die Student*innen das Online-Angebot als Mehrwert an. Bei der objektiven Evaluation des Lernerfolgs, zeigte sich kein wesentlicher Unterschied zwischen dem Online-Untersuchungskurs und der Präsenzlehre – auch wenn die Ergebnisse in beiden Fällen sehr positiv ausfallen und das Notenspektrum nicht vollständig ausgereizt wurde.

Ein Online-Untersuchungskurs kann das Lehrportfolio ergänzen. Erste E‑Learning-Angebote wurden an unserer Klinik im Jahr 2006 geschaffen [15]. Schon damals war die Rückmeldung der Student*innen positiv, jedoch bemängelte damals ein Drittel der Student*innen technische Probleme insbesondere bei den Videos. Beim neu entwickelten Online-Untersuchungskurs traten derartige Probleme deutlich seltener auf, was vermutlich mit der zunehmenden Verbreitung schneller Internetverbindungen zusammenhängt. Die Verfügbarkeit der Bandbreitenklasse ≥ 50 Mbit/s in Deutschland wuchs allein von 39,5 % im Jahr 2010 auf 90,2 % im Jahr 2019 an [19, 20]. Auch die Kameratechnik und die Software zur Nachbearbeitung von Videos wurden weiterentwickelt, und die Videoproduktion wurde vereinfacht.

Eine Mehrheit von 71 % der befragten Student*innen wünschte sich eine Kombination aus Digital- und Präsenzlehre für die Zukunft. So sind in den kommenden Semestern hybride Formate im Sinne der Flipped-Classroom-Methode denkbar, bei denen die Vorbereitung der theoretischen Inhalte bereits online von zu Hause aus erfolgt und somit mehr Zeit für die Vertiefung und praktischen Übungen vor Ort zur Verfügung steht. Zusätzlich könnten die „Fragen zum Selbststudium“ statt am Ende des Skripts in Form eines Online-Quiz bereitgestellt werden.

Auch in der Ausbildung von Assistenzärzt*innen bietet ein Online-Kurs spannende Optionen. Beispielsweise kann eine Überlagerung von Operationsvideos mit zusätzlichen Informationen die chirurgische Ausbildung bereichern [2, 6, 10, 13, 14, 22].

Durch die Digitalisierung kann der Fachbereich für zukünftige Ärzt*innen möglicherweise auch attraktiver werden und eine Vertiefung durch fakultative Materialien für besonders interessierte Student*innen erlauben. Der in dieser Studie festgestellte Anteil von 25 % der Student*innen, die sich das Fach Augenheilkunde als berufliche Perspektive vorstellen konnten, ist im Vergleich zum „Berufsmonitoring Medizinstudenten“ der Kassenärztlichen Bundesvereinigung 2018 ein hoher Wert (6 % von 13.334 Student*innen) [21]. Nach dem Online-Untersuchungskurs lag damit eine überdurchschnittliche Offenheit für eine berufliche Perspektive in der Ophthalmologie vor – auch wenn der Zeitpunkt der Befragung direkt nach dem Kurs möglicherweise zu anderen Schlüssen führt als eine Befragung am Ende des Studiums.

### Limitationen

Im Gegensatz zur anonymen und maschinellen Auswertung der Abrufzahlen der Videos und der Prüfungsergebnisse ist die Evaluation eine Selbsteinschätzung und unterliegt damit verschiedenen Faktoren. Die Rücklaufquote von 41 % in dieser Studie ist niedrig, jedoch mit anderen Studien vergleichbar [1, 3]. Eine häufig diskutierte Ursache kann eine „Evaluationsmüdigkeit“ aufgrund zahlreicher Evaluationsaufforderungen zum Ende des Semesters sein. Eine Lösung könnte darin bestehen, jede Veranstaltung von einer kleineren Stichprobe zufällig ausgewählter Teilnehmender evaluieren zu lassen [11]. Alternativ wäre eine Zwischenevaluation während des Semesters denkbar, um Änderungswünsche direkt adressieren zu können, dies hätte in unserem Fall jedoch die wissenschaftliche Auswertbarkeit erschwert. Da gute Leistungen potenziell mit einer erhöhten Bereitschaft zur Teilnahme an Lehrevaluationen einhergehen [1], können die Evaluationsergebnisse möglicherweise positiver ausgefallen sein. Die Vergleichbarkeit der Prüfungsergebnisse ist möglicherweise eingeschränkt, da aus Sicherheitsgründen nur 3 statt bisher 4 Prüfungsstationen geprüft wurden.

## Fazit für die Praxis

- Ein Online-Untersuchungskurs kann relevante Inhalte des praktischen ophthalmologischen Untersuchungskurses vermitteln.
- Student*innen schätzen den persönlichen Kontakt und die Interaktionsmöglichkeit einer Präsenzveranstaltung.
- Ein Online-Untersuchungskurs bietet Möglichkeiten, praktisches Wissen und Fähigkeiten zu vermitteln.
- Die manuelle Auseinandersetzung mit den Untersuchungsinstrumenten sollte auch im Rahmen eines Online-Untersuchungskurses ermöglicht werden.
- Zukünftige Lernformate können von einer Kombination aus Online- und Praxisangeboten profitieren.

## Abkürzungen

COVID-19: Coronavirus-Disease-2019
DOG: Deutsche Ophthalmologische Gesellschaft
OSCE: Objective structured clinical examination
SD: Standardabweichung
